# Improving the Output Efficiency of Triboelectric Nanogenerator by a Power Regulation Circuit

**DOI:** 10.3390/s23104912

**Published:** 2023-05-19

**Authors:** Wenbo Li, Baichuan Leng, Shengyu Hu, Xiaojun Cheng

**Affiliations:** 1 Beijing Institute of Nanoenergy and Nanosystems, Chinese Academy of Sciences, Beijing 101400, China; liwenbo@binn.cas.cn (W.L.); lengbaichuan@binn.cas.cn (B.L.); hushengyu@binn.cas.cn (S.H.); 2 School of Nanoscience and Technology, University of Chinese Academy of Sciences, Beijing 100049, China

**Keywords:** triboelectric nanogenerator, power regulation circuit, performance improvement, conduction time, two-stage output

## Abstract

Triboelectric nanogenerator (TENG) is a promising technology for harvesting energy from various sources, such as human motion, wind and vibration. At the same time, a matching backend management circuit is essential to improve the energy utilization efficiency of TENG. Therefore, this work proposes a power regulation circuit (PRC) suitable for TENG, which is composed of a valley-filling circuit and a switching step-down circuit. The experimental results indicate that after incorporating a PRC, the conduction time of each cycle of the rectifier circuit doubles, increasing the number of current pulses in the TENG output and resulting in an output charge that is 1.6 fold that of the original circuit. Compared with the initial output signal, the charging rate of the output capacitor increased significantly by 75% with a PRC at a rotational speed of 120 rpm, significantly improving the utilization efficiency of the TENG’s output energy. At the same time, when the TENG powers LEDs, the flickering frequency of LEDs is reduced after adding a PRC, and the light emission is more stable, which further verifies the test results. The PRC proposed in this study can enable the energy harvested by the TENG to be utilized more efficiently, which has a certain promoting effect on the development and application of TENG technology.

## 1. Introduction

Energy is an indispensable factor for social development. With the increasing severity of the energy crisis and the increasing requirements for environmental protection, the demand for sustainable and efficient energy conversion and storage technologies is becoming more and more urgent [1,2,3]. In recent years, the innovation of new energy and the utilization of environmental energy have been research hotspots, which are necessary for energy storage and optimization of related renewable energy [4,5]. As a promising energy harvesting technology, triboelectric nanogenerator (TENG) has attracted considerable attention due to its sustainability and low cost [6,7], as it has been proven capable of harvesting energy from various sources, including human motion, wind, vibration and water currents, which makes it highly versatile in practical applications [8,9,10,11,12]. At the same time, the application directions of TENG are also very wide, covering smart phones, wearable devices, wireless sensor networks, fitness equipment and other fields [13]. It also has broad application prospects in environmental monitoring, intelligent transportation, smart home, medical and health fields [14,15].

Despite its well-established theoretical basis and significant advantages, the TENG faces several challenges that hinder its efficiency [16,17]. Furthermore, stability remains a significant problem in practical applications [18,19,20]. Therefore, in-depth research on the performance and mechanism of TENG is essential for promoting its practical application [21,22]. Scholars have explored and conducted experimental research, including theoretical analysis and derivation, material selection and preparation, structure design and optimization, as well as performance testing and analysis, to address the challenges related to the TENG [23,24,25,26,27]. In addition to studying TENG’s characteristics, developing a power management circuit that matches the output of TENG is crucial to improving its efficiency [28,29]. Hu et al. reported a simple and adjustable automatic spark switch circuit, which achieved energy accumulation and rapid release [30]. Cheng et al. proposed a dual-loop circuit, reducing the energy loss in the diode to 47.1% [31]. Zhang et al. demonstrated a universal power management circuit that achieved an efficiency of 80% in matching the impedance of a TENG [32]. A power management circuit can convert the TENG’s alternating current (AC) output into a direct current (DC) output, which can power electronic devices or store energy in batteries or capacitors [33,34]. Maximizing harvested energy by increasing energy conversion efficiency is a significant challenge in TENG technology [35]. A suitable power management circuit can effectively enhance the TENG’s output performance, playing a vital role in promoting its practical application.

In this paper, a power regulation circuit (PRC) suitable for TENG is proposed, which is composed of a first-stage valley-filling circuit and a second-stage switching step-down circuit, and its electrical performance is tested by a typical freestanding-mode TENG. Unlike common power management circuits that use switch circuits to reduce the duty cycle of the TENG, a PRC increases the conduction time of the TENG to increase the output charge and improve the energy utilization efficiency of the TENG. The experimental results show that adding the PRC increases the power supply time of the TENG in each cycle, thereby increasing the pulse number of the TENG output signal and the output charge is 1.6-fold higher than the initial value. Compared with the initial output signal, the charging rate of the output capacitor increases by 75%, improving the TENG’s performance significantly. The stability of the PRC is further proved by frequency conversion charging experiments. At the same time, when the TENG supplies power to LEDs with a motion frequency of 2 Hz, the LEDs using the PRC flicker less than LEDs powered by direct rectification, the light is also more stable, further verifying the test results. The proposed PRC can efficiently utilize the output energy of the TENG, providing new insights into the storage and utilization of the TENG’s energy.

## 2. Structure and Mechanism

**Figure 1:**
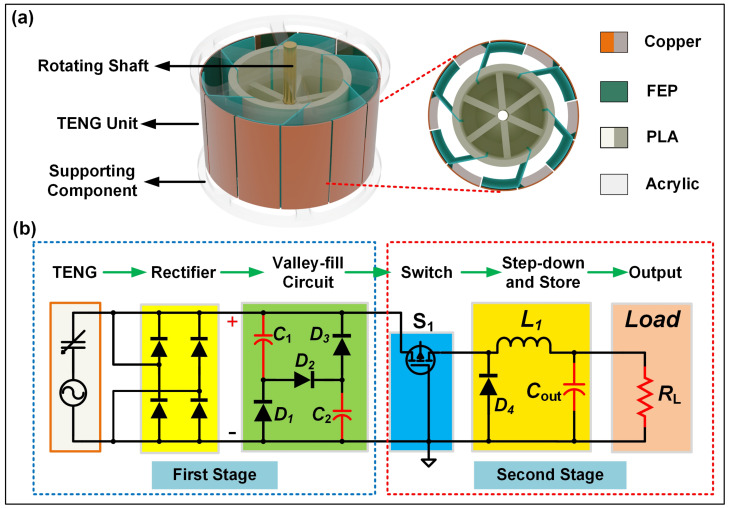
Design schematic diagram of the TENG and the PRC. (**a**) System structure of the TENG. (**b**) The circuit diagram of the PRC.

Figure 1 presents a schematic diagram of a TENG and a PRC system, where Figure 1a illustrates the specific structure of the TENG. As the power supply segment of the PRC, the TENG is used to verify the applicability of the PRC for the TENG’s power management and compared with the signal output after through the PRC. The TENG adopts a rotating freestanding mode, comprising a rotor, a shell and a shaft. The shell structure is made of an acrylic tube with a diameter of 94 mm, with six pairs of copper electrodes attached to its inner wall. The rotor is made of polylactide (PLA) material and uniformly inlaid with six pieces of fluorinated ethylene propylene (FEP) films, forming the power generation unit of the TENG. The TENG is driven by a rotating motor, and the output signals of the system are measured by a programmable electrometer and data acquisition system, which are processed and stored by LabVIEW software and computer. Figure 1b shows the schematic diagram of the PRC system, which consists of two-stage circuits. The first stage comprises a rectifier and a valley-filling circuit to increase the conduction time of the rectifier bridge during the TENG’s unit motion cycle and enhance the initial output energy. The second-stage circuit steps down and stores the previous stage circuit to better match the output load. A PMOS transistor is used as a control switch, automatically turned on and off based on the magnitude of the first-stage output signal. To facilitate the experiment’s debugging and component parameter determination, a PRC system was built on a breadboard, acting as a passive power management circuit directly connected to the TENG’s output.

**Figure 2:**
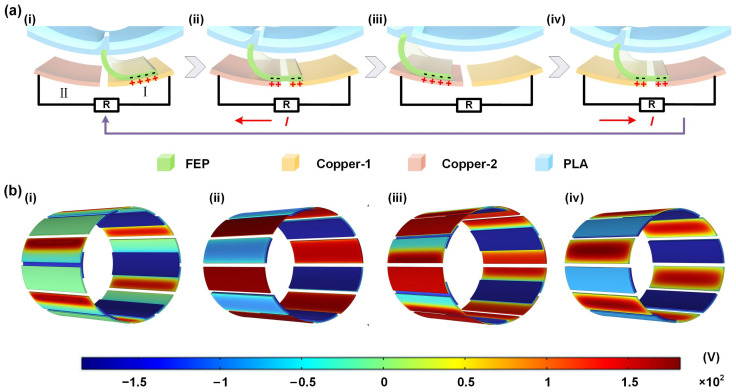
Principle analysis of the TENG: (**a**) The specific working principle of the TENG, (**i**–**iv**) the charge transfer relationship between the FEP film and the copper electrode when the TENG rotates; (**b**) (**i**–**iv**) simulation diagram of charge distribution in different states of the TENG.

Figure 2 depicts the specific working principle of the TENG, which mainly comprises four processes. As presented in Figure 2a, according to the triboelectric sequence table, the FEP film surface easily gains electrons, resulting in a negative surface potential. Conversely, the copper electrode surface readily loses electrons, leading to a positive surface potential. In the initial state (Figure 2a(i)), the rotor remains stationary, and the FEP film is in full contact with copper electrode I. Consequently, contact electrification causes charge transfer, and both the copper electrode and the FEP film acquire equal amounts of positive and negative charges. When the FEP film is rotated to the state illustrated in Figure 2a(ii), it contacts both copper electrode I and copper electrode II, inducing electrostatic induction that drives electrons on electrode II towards electrode I, resulting in a secondary electrode under external load, and the current from electrode I to electrode II. As the rotor continues to move to the state displayed in Figure 2a(iii), the FEP film comes into complete contact with copper electrode II, and electrostatic equilibrium is reached, with no charge flowing. Subsequently, the rotor rotates to state iv (Figure 2a(iv)), under external load conditions, and a potential difference drives the generation of current in the opposite direction to the previous half-cycle, eventually returning to the initial state, initiating a new cycle. Therefore, continuous rotation of the TENG produces a continuous AC signal. Additionally, COMSOL was employed to simulate the electrostatic field, exhibiting the power generation mechanism of the TENG under different motion states (Figure 2b).

**Figure 3:**
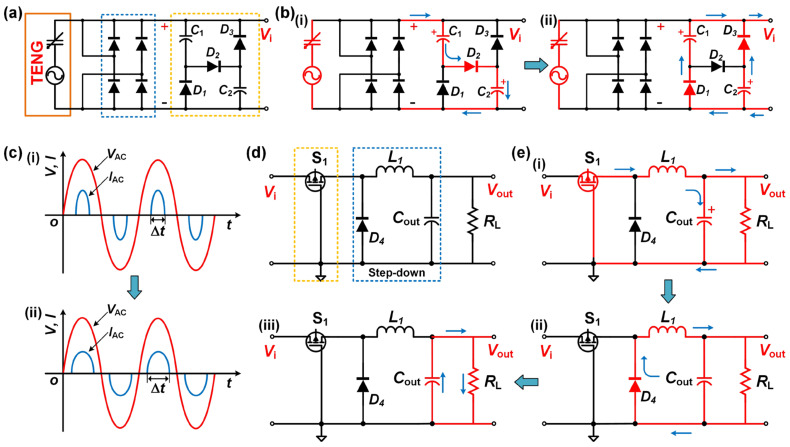
PRC-specific workflow: (**a**) the circuit diagram of the first stage in the PRC system; (**b**) the workflow of the first-stage circuit in the PRC, (**i**) series charging and (**ii**) parallel discharging of capacitors.; (**c**) the output mechanism diagram of the first-stage circuit in the PRC; (**d**) the circuit diagram of the second stage in the PRC system; (**e**) (**i**–**iii**) the workflow of the second-stage circuit within one cycle in the PRC.

Figure 3 illustrates the specific working process of the PRC. Figure 3a represents the first-stage circuit, which comprises a rectifier and a passive valley-filling circuit. To illustrate the functioning of the first-stage circuit, Figure 3b presents its detailed working principle. When the voltage of the TENG is high, the output current signal follows the path illustrated in Figure 3b(i). The current charges *C*_1_ and *C*_2_, and *D*_2_ conducts while *D*_1_ and *D*_3_ are cut off. In contrast, when the voltage is low, the capacitors *C*_1_ and *C*_2_ are discharged through *D*_1_ and *D*_3_ while *D*_2_ is cut off, as shown in Figure 3b(ii). Meanwhile, when selecting a capacitor, it must be ensured that the values of *C*_1_ and *C*_2_ are equal, and in order to match the internal resistance of the TENG, the selection of the capacitor should not be too large. The selected capacitor value is 0.1 μF. Furthermore, neglecting the voltage drop across the diodes, when the TENG output voltage attains V_m_, the voltage drops across *C*_1_ and *C*_2_ are both 1/2 V_m_. Subsequently, when the output voltage of the TENG drops to 1/2 Vm, the rectifier bridge diode is cut off. At this point, *D*_1_ and *D*_3_ conduct, and the two capacitors are connected in parallel to power the load. Compared to the basic rectifier circuit, the power supply after rectification needs to be higher than V_m_ to provide power to the external load. Therefore, as shown in Figure 3c, compared to the case of directly rectifying the output (Figure 3c(i)), the first-stage circuit increases the power supply time of the external load within one cycle, and due to the longer conduction time, only a small peak current is required to fulfill the power requirement within one cycle (Figure 3c(ii)). The second-stage output circuit, as illustrated in Figure 3d, comprises a PMOS switch and a step-down storage section. Figure 3e demonstrates the specific working process. Initially, when the output voltage Vi of the first stage reaches the threshold voltage of the switch S_1_, that is, when the source voltage of the PMOS transistor is 2 to 3 V higher than the gate voltage, the energy is transferred from the first-stage circuit to the second-stage circuit, and S_1_ is passed through the change in the voltage is automatically turned on and off, and assists the second-stage circuit to realize the voltage reduction, realizing a regulation process of the passive switch. The output current powers the capacitor *C*_out_ and the load via the inductor *L*_1_, as depicted in Figure 3e(i). Conversely, when the voltage falls below *V*_i_, the switch S_1_ is tuned off, causing the energy stored in the inductor to be released to *C*_out_ and the load. At this point, *D*_4_ is turned on, creating a closed loop, as shown in Figure 3e(ii). Ultimately, when the energy stored in *C*_out_ reaches a certain threshold, it becomes capable of supplying stable power to the output load, as displayed in Figure 3e(iii). By employing the aforementioned two-stage circuits, the PRC system can accomplish the entire working process.

## 3. Results and Discussion

### 3.1. Basic Output Performance

**Figure 4:**
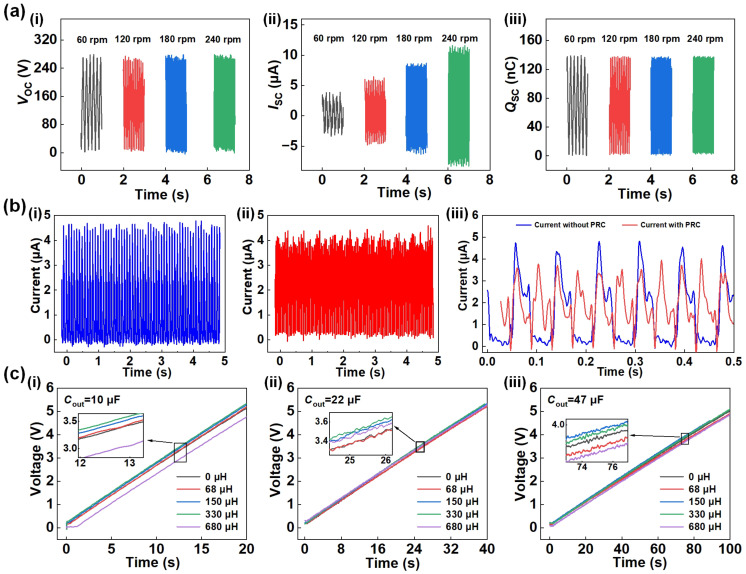
Basic output characteristics of the TENG and the PRC. (**a**) (**i**–**iii**) The original signals of voltage, current and charge of the TENG at different frequencies; (**b**) (**i**) short-circuit current output by direct rectification and (**ii**) by the PRC of the TENG, (**iii**) amplification of the current signal for comparison.; (**c**) (**i**–**iii**) influence of different inductance values on charging characteristics of output capacitor.

Figure 4 illustrates the fundamental performance tests of the TENG and the PRC. Specifically, Figure 4a(i–iii) exhibit the open-circuit voltage, short-circuit current, and transferred charge output signals of the TENG at different rotational speeds, respectively. Notably, the peak values of the open-circuit voltage and transferred charge remain nearly constant with increasing rotational frequency, whereas the short-circuit current gradually increases, having established the essential output performance and stable operating state of the TENG. Figure 4b(i,ii), respectively, demonstrate the short-circuit currents outputted by the TENG rectification and the TENG integrated with a first-stage circuit, at a consistent rotational speed of 120 rpm (unless stated otherwise, all subsequent experimental conditions were conducted at this rotational speed). The current pulse signals in Figure 4b(ii) appear to be more closely packed. To further elucidate the effect of the first-stage circuit in the PRC on the TENG output signal, Figure 4b(iii) compares the two sets of current signals in Figure 4b(i,ii) with amplification. It can be concluded that after the first-stage circuit, the number of pulse currents output by the TENG per unit time doubled, and the transferred charge amount increased by 60%. Although the peak current value slightly decreases, the accumulated energy output during the entire cycle shows a significant increase, which indicates that the first-stage circuit effectively increases the conduction time of the rectifier bridge diode of the TENG, leading to improved output power. It is worth noting that in the PRC system, the inductance *L*_1_ plays a critical role in determining the circuit performance, particularly when the frequency of TENG motion is relatively high and the switch is rapidly turned on and off. To investigate this effect, experiments were carried out using different inductance values, as shown in Figure 4c(i–iii). *C*_out_ with values of 10 μF, 22 μF, and 47 μF was used to test the charging time. Since the motion frequency of the TENG itself is not high, μH level inductance values are chosen. The capacitance charging curves show that the charging rate of *C*_out_ increases with an increasing inductance value. However, when the value of *L*_1_ exceeds 330 μH, the delay caused by inductance becomes more significant, leading to a reduction in the charging rate. Based on our results, the optimal value of *L*_1_ is between 150 μH and 330 μH.

### 3.2. Demonstration of the PRC

**Figure 5:**
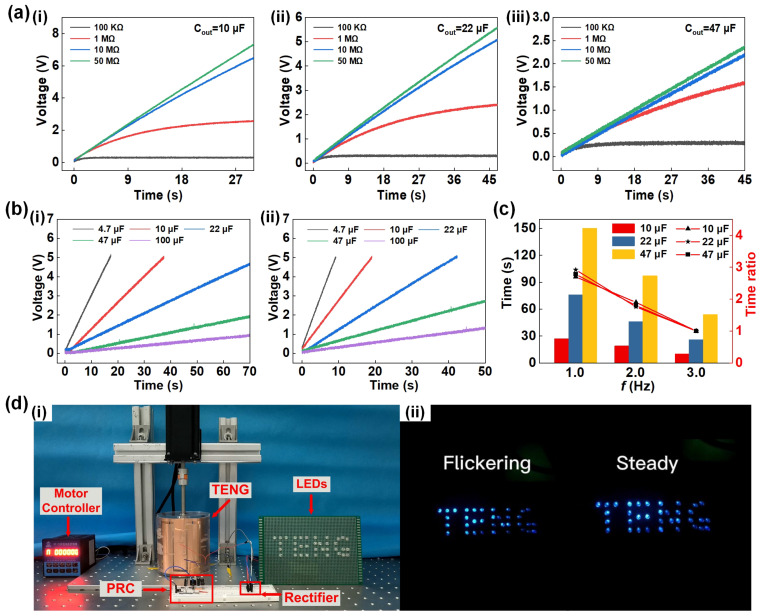
The output performance test and application demonstration of the PRC. (**a**) (**i**–**iii**) Curves of the charging voltage and the charging rate of *C*_out_ under different loads; (**b**) comparison experiment of output capacitor charging (**i**) with PRC and (**ii**) without the PRC; (**c**) the relationship between frequency and charging time; (**d**) (**i**) experimental test physical diagram and (**ii**) application demonstration of the TENG powering LEDs in two output modes.

Once the basic principles and parameters of the PRC system are determined, Figure 5 presents the final output performance analysis of the PRC system, along with comparative experiments and application demonstrations. Figure 5a shows the load charging characteristic curves for different *C*_out_ of 10 µF, 22 µF, and 47 µF, indicating that the internal resistance of the TENG should be considered when choosing the load. If the *C*_out_ is insufficiently large, the load voltage will fluctuate, leading to poor load-bearing capacity. Moreover, when the capacitance of *C*_out_ remains constant, the charging speed of *C*_out_ will be faster with a larger load. Comparative experiments were conducted as shown in Figure 5b(i,ii), where capacitors were charged under two different output conditions, one with the PRC system and the other without it. The results demonstrate that the charging rate of capacitors increased by 75% when the capacitor is charged to 5 V with the PRC system included. In addition, the charging time is significantly reduced with the inclusion of the PRC system. In order to verify the stability of the PRC system, Figure 5c presents a bar chart of the charging time of three groups of output capacitors charged to 5 V under different TENG motion frequencies. The charging time ratios of the three groups of capacitors are also tested at different frequencies using 3 Hz as the reference frequency, as shown in the curve in Figure 5c, the three curves almost coincide and can be fitted into one curve, indicating that the charging time varies proportionally with the motion frequency, and the entire circuit system is very stable. Finally, Figure 5d demonstrates the actual application capability of the PRC circuit through an experimental demonstration. Figure 5d(i) shows the testing system diagram, and the power supply of LEDs under two output modes, direct rectification and PRC output, is tested at a TENG speed of 120 rpm. The experimental findings indicate that when the LEDs are connected to the PRC (as illustrated in Figure 5d(i) and Video S1 in the Supplementary Materials), the flashing phenomenon is reduced and the overall lighting process becomes more stable. These demonstration results are consistent with the test data, thus confirming that the PRC can enhance the output efficiency of the TENG and improve the output stability.

## 4. Conclusions

In summary, the PRC system increases the power supply time of the TENG in each cycle, thereby increasing the number of pulses in the TENG output current signal. As a result, the total output energy per unit cycle increases, and compared to the initial output signal, the charging rate of the output capacitor increases by 75% after the PRC is added, improving the output efficiency of the TENG significantly. The PRC consists of a first-stage valley-filling circuit and a second-stage switching step-down circuit. The circuit performance was tested by a rotation-type freestanding-mode TENG. The frequency conversion charging experiment further demonstrated the stability of the PRC. At the same time, when the TENG supplies power to the LEDs at 120 rpm, the LEDs using the PRC flicker less and emit more stable light than the LEDs supplied by direct rectification, further validating the test results. The PRC proposed in this study can more effectively harvest and utilize the TENG’s energy, which has a certain promoting effect on the storage and utilization of the output energy of the TENG.

## Data Availability

Not applicable.

## Supplementary Materials

The following are available online at https://www.mdpi.com/article/10.3390/s23104912/s1, Video S1: Demonstration of lighting LEDs by the TENG with and without a PRC.

## Nomenclature

TENG	Triboelectric Nanogenerator
PRC	Power Regulation Circuit
PLA	Polylactide
FEP	Fluorinated Ethylene Propylene
PMOS	Positive channel Metal Oxide Semiconductor
*C*_1_, *C*_1_	Capacitance of the first-stage circuit
*D*_1_, *D*_2_, *D*_3_, *D*_4_	Diode
*L*_1_	Inductance
*C*_out_	Output Capacitance
*R*_L_	Load
*V*_i_	Input Voltage
*V*_out_	Output Voltage
